# The Role of Radiomics in the Prediction of Clinically Significant Prostate Cancer in the PI-RADS v2 and v2.1 Era: A Systematic Review

**DOI:** 10.3390/cancers16172951

**Published:** 2024-08-24

**Authors:** Andreu Antolin, Nuria Roson, Richard Mast, Javier Arce, Ramon Almodovar, Roger Cortada, Almudena Maceda, Manuel Escobar, Enrique Trilla, Juan Morote

**Affiliations:** 1 Department of Radiology, Institut de Diagnòstic per la Imatge (IDI), Hospital Universitari Vall d’Hebron, 08035 Barcelona, Spain; nuria.roson.idi@gencat.cat (N.R.); javier.arce.idi@gencat.cat (J.A.); 2 Department of Surgery, Universitat Autònoma de Barcelona, 08193 Bellaterra, Spain; enrique.trilla@vallhebron.cat (E.T.); juan.morote@uab.cat (J.M.); 3 Department of Radiology, Hospital Universitari Vall d’Hebron, 08035 Barcelona, Spain; richard.mast@vallhebron.cat (R.M.); ramon.almodovar@vallhebron.cat (R.A.); roger.cortada@vallhebron.cat (R.C.); manel.escobar@vallhebron.cat (M.E.); 4 Vall d’Hebron Research Institute, 08035 Barcelona, Spain; almudena.maceda@vhir.org; 5 Department of Urology, Vall d’Hebron University Hospital, 08035 Barcelona, Spain

**Keywords:** clinically significant prostate cancer, PI-RADS, magnetic resonance imaging, radiomics, deep learning, machine learning, systematic review, prediction

## Abstract

**Simple Summary:**

There is still an overdiagnosis of indolent prostate cancer (iPCa) lesions using the Prostate Imaging-Reporting and Data System (PI-RADS), and radiomics has emerged as a promising tool to improve the diagnosis of clinically significant prostate cancer (csPCa) lesions. However, the current state and applicability of radiomics remains a challenge. This systematic review aims at evaluating the evidence of handcrafted and deep radiomics in differentiating lesions at risk of having csPCa from those with iPCa and benign pathology. The review highlighted a good performance of radiomics but without significant differences with radiologist assessment (PI-RADS), as well as several methodological limitations in the reported studies, which might induce bias. Future studies should improve methodological aspects to ensure the clinical applicability of radiomics, especially the need for clinical prospective studies and the comparison with PI-RADS.

**Abstract:**

Early detection of clinically significant prostate cancer (csPCa) has substantially improved with the latest PI-RADS versions. However, there is still an overdiagnosis of indolent lesions (iPCa), and radiomics has emerged as a potential solution. The aim of this systematic review is to evaluate the role of handcrafted and deep radiomics in differentiating lesions with csPCa from those with iPCa and benign lesions on prostate MRI assessed with PI-RADS v2 and/or 2.1. The literature search was conducted in PubMed, Cochrane, and Web of Science databases to select relevant studies. Quality assessment was carried out with Quality Assessment of Diagnostic Accuracy Studies 2 (QUADAS-2), Radiomic Quality Score (RQS), and Checklist for Artificial Intelligence in Medical Imaging (CLAIM) tools. A total of 14 studies were deemed as relevant from 411 publications. The results highlighted a good performance of handcrafted and deep radiomics methods for csPCa detection, but without significant differences compared to radiologists (PI-RADS) in the few studies in which it was assessed. Moreover, heterogeneity and restrictions were found in the studies and quality analysis, which might induce bias. Future studies should tackle these problems to encourage clinical applicability. Prospective studies and comparison with radiologists (PI-RADS) are needed to better understand its potential.

## 1. Introduction

Prostate cancer (PCa) is the most frequent malignant tumor diagnosed in men, and the second cause of cancer-related death among men [1]. The modified Gleason Score is the recommended PCa grading, and it is based on the microscopic patterns seen in sample tissues obtained from prostate biopsies, ranging from 6 (better prognosis) to 10 (worse prognosis). In 2014, the ISUP Gleason Grading Conference on Gleason Grading of PCa introduced grade groups to better stratify men with PCa, ranging from 1 to 5. ISUP grade 1 (equivalent to Gleason Score of 6) carcinomas have a better prognosis than ISUP grade > 1 (equivalent to Gleason Score of 7 or above) carcinomas [2]. Men with ISUP grade 1 PCa have a better prognosis and can benefit from active surveillance programs in the right conditions, while men with ISUP grade > 1 PCa tend to require curative treatment and follow-up. Consequently, PCa can be further divided into indolent PCa (iPCa), which has an ISUP grade 1, and clinically significant PCa (csPCa), which has an ISUP grade > 1. Risk-stratified PCa screening focuses on improving early detection of csPCa and reducing the overdetection of iPCa, thus avoiding unnecessary prostate biopsies and related side effects [3,4,5].

Much of the progress in the early detection of csPCa comes from multiparametric or biparametric prostate magnetic resonance imaging (mpMRI or bpMRI) performed before prostate biopsy, which allows the identification of suspicious lesions and the estimation of a semiquantitative risk of csPCa through the Prostate Imaging-Report and Data System (PI-RADS), currently in its version 2.1 [6]. The indication for prostate biopsy is established when the PI-RADS is ≥3 since the negative predictive value of MRI when using PI-RADS 2.1 ranges between 96% and 98% for PI-RADS 1 and 2, respectively. The positive predictive value of PI-RADS 3 is 20%, that of PI-RADS 4 is 52%, and that of PI-RADS 5 is 89% [7,8]. Moreover, MRI increases sensitivity for the detection of csPCa by enabling targeted biopsies of suspicious lesions, although it is complemented by the classic systematic biopsy since a small percentage of csPCa is found only in this type of biopsy [9]. Such is the evidence that the European Union recommends PCa screening based on serum prostate-specific antigen (PSA) and MRI [3]. Therefore, the current approach is to perform an MRI in men with a serum PSA > 3.0 ng/mL and/or an abnormal digital rectal examination (DRE), followed by a targeted biopsy of PI-RADS ≥3 lesions, complemented with a systematic prostate biopsy [10]. Even though the paradigm of early diagnosis has radically changed thanks to the introduction of MRI, there are still limitations in the application of the latest PI-RADS version [11]. Moreover, there is still important inter-reader variability when assessing prostate lesions using PI-RADS version 2 and 2.1 [12,13], and the overdiagnosis of iPCa in PI-RADS 3 lesions remains a challenge [8]. Consequently, there is a need for new biomarkers and csPCa predictive models to reduce the number of false positives [14].

Radiomics is the extraction of quantitative imaging features from radiological images that are imperceptible to radiologists with the use of specific artificial intelligence (AI) software. These mineable high-dimensional data maximize the information that can be extracted from medical images, as a diagnostic tool or even as prognostic one to improve clinical decisions in the context of personalized precision medicine [15]. Traditional defined and well-known quantitative features known as handcrafted radiomics have been widely used in medical imaging [15]. However, the inception of deep learning algorithms has allowed the automatic extraction of new unknown quantitative features, known as deep radiomics, which might overcome the classical approach [16].

Radiomics has shown promising results in computed tomography (CT) and MRI for improving PCa detection, PCa risk-group classification, risk of biochemical recurrence, and risk of metastatic disease, as well as the identification of extra-prostatic extension or even the evaluation of treatment toxicity, among others [17]. The discrimination between csPCa and iPCa is the main field of research in radiomics applied to PCa [17,18] due to the current diagnostic limitations previously highlighted. A radiomic or multivariable model capable of improving the prediction of PI-RADS in detecting csPCa might help in reducing the number of false positives and unnecessary biopsies in men with iPCa.

Due to this, the European Society of Urogenital Radiology (ESUR) and European Association of Urology (EAU) have advocated for developing robust AI models to overcome these limitations [19]. However, there is still limited evidence of the role of radiomics in real clinical scenarios, as well as its role in predictive models using other clinical variables and the comparison with the PI-RADS.

The main aim of this systematic review is to evaluate the current evidence of the role of handcrafted and deep radiomics in differentiating lesions with csPCa from those with iPCa and benign lesions on prostate MRI assessed with PI-RADS v2 and/or 2.1. Secondary objectives include the comparison between radiomic models and radiologists reporting through the latest PI-RADS versions, as well as the performance in predictive models when combined with other clinical variables.

## 2. Evidence Acquisition

### 2.1. Literature Search

The search was conducted in PubMed, Cochrane, and Web of Science databases to select relevant studies for assessing the aims of this review which were published before 30 April 2024. The Boolean strings and keywords used in the search were (Radiomic OR Machine Learning OR Deep Learning) AND Clinically Significant Prostate Cancer AND (Magnetic Resonance Imaging OR PI-RADS). Two independent reviewers, A.A. and J.M., double-blind-reviewed the retrieved reports according to the eligibility criteria. In case of disagreement, consensus was achieved by mutual accordance between both reviewers. References of selected articles were also manually reviewed for additional citations. The Preferred Reporting Items for Systematic Reviews and Meta-analyses (PRISMA) criteria were followed for conducting this systematic review [20]. This systematic review was registered in PROSPERO (International Prospective Register of Systematic Reviews), with the ID number CRD42024527768. A narrative synthesis was chosen for this systematic review due to the heterogeneity of the selected studies.

### 2.2. Eligibility Criteria

The eligible studies were selected according to inclusion criteria based on the Population, Intervention, Comparator, Outcome (PICO) framework [21], with the detailed breakdown depicted in Supplementary Table S1. The inclusion criteria derived from PICO were (i) men with suspected PCa with consequent evaluation with prostatic mpMRI or bpMRI; (ii) retrospective or prospective assignment of prostatic lesions with PI-RADS v2 or v2.1; (iii) targeted +/− systematic biopsy or radical prostatectomy performed after the mpMRI or bpMRI; (iv) diagnosis of PCa based on histopathological findings, defining csPCa as International Society of Urogenital Pathology (ISUP) grade group > 1 and iPCa as ISUP grade group 1 [2]; (v) outcome measured as diagnostic performance of a handcrafted or deep radiomics model for differentiating csPCa from iPCa and benign lesions with a measurable metric: area under the curve (AUC), sensitivity, specificity, accuracy, positive predictive value (PPV), and negative predictive value (NPV). Exclusion criteria were (i) men in active surveillance or with prior prostate cancer treatment (if specified in the methodology); (ii) studies derived from public datasets (excluding external validation sets). Men with only systematic biopsies were incorporated if no positive findings were detected in bpMRI or mpMRI.

Observational studies were included in this review due to the current lack of randomized clinical trials using AI in clinical settings. Systematic reviews, meta-analyses, letters, conference abstracts and unpublished manuscripts were excluded. In the case of different studies using the same population or datasets, the best methodological study was selected and the rest were discharged. Studies not written in English were excluded.

### 2.3. Quality Assessment

Risk of bias assessment was analyzed with the Quality Assessment of Diagnostic Accuracy Studies 2 (QUADAS-2) tool [22]. The risk was evaluated by two independent reviewers (A.A. and J.M.) as unclear, low, or high. In case of disagreement, consensus was achieved by mutual accordance between both reviewers. If all the domains were regarded as low risk, the study was given a low risk of bias. If the study had one or more unclear risk of bias, it was considered as an unclear risk of bias. If the study contained any high-risk domain, it was considered as having a high risk.

### 2.4. Artificial Intelligence Quality Assessment

In addition to the QUADAS-2 [22] risk of bias assessment, each study was also reviewed with specific AI-quality standards guidelines. For studies using handcrafted radiomics and traditional machine learning (ML) methods, the quality was evaluated using the Radiomics Quality Score (RQS), giving a score out of 36 points for each paper included [15]. The RQS v2.0 was not used since it was still under development at the time of this systematic review. Studies using deep radiomics were assessed using the Checklist for Artificial Intelligence in Medical Imaging (CLAIM) [23]. The 42-item checklist of this guideline was evaluated in each case, regarded as fulfilled or not. The 2024 update was not available at the time of this systematic review.

### 2.5. Data Collection

The data to be extracted were agreed upon between A.A. and J.M. before the beginning of the extraction, detailed in Supplementary File S2. Both authors were responsible for data collection of the studies included. A tabular structure was used to display the results of individual studies, referenced based on author and year of publication. A comprehensive synthesis of the main findings based on each table was then performed, adding extra information not included in the tables.

## 3. Evidence Synthesis

### 3.1. Study Selection

**Figure 1:**
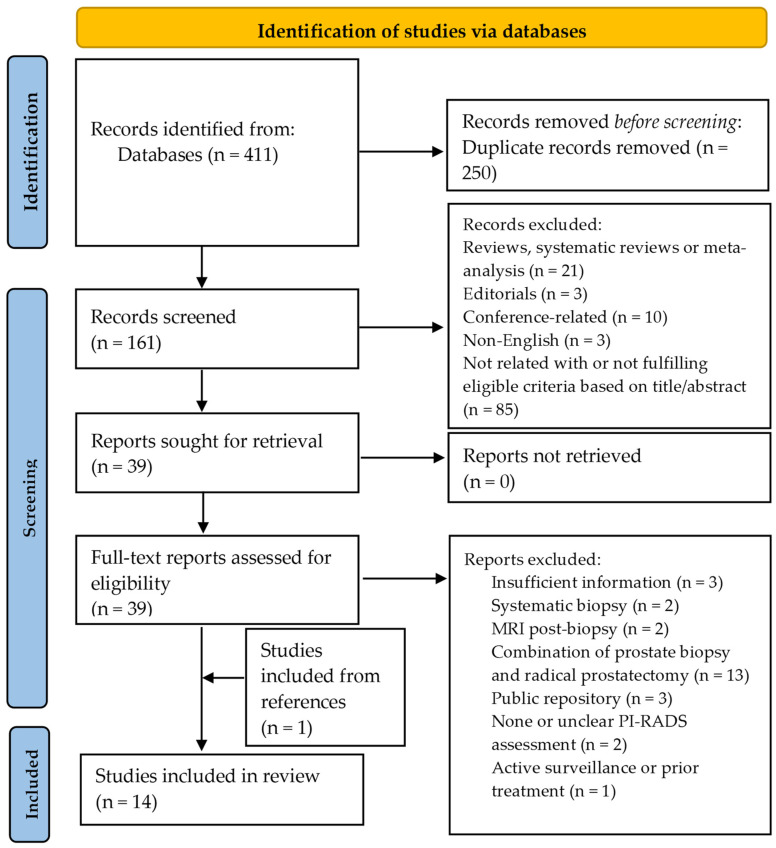
Preferred Reporting Items for Systematic Reviews and Meta-Analysis (PRISMA 2020) flow diagram for the selection of relevant studies based on the search strategy.

A total of 411 titles were obtained according to the search strategy, and 250 were excluded because of duplicates. The remaining 161 were analyzed based on the title and abstract, and 39 were deemed as relevant. A total of 21 reviews, systematic reviews, and meta-analyses were discarded, as well as three editorials and 10 conference-related papers. Three articles were written in a different language than English and were also discarded. The full texts were finally reviewed for definite inclusion, with a final number of 13 studies fulfilling the required criteria. An extra study was incorporated from the references of the analyzed papers, for a total of 14 selected studies [24,25,26,27,28,29,30,31,32,33,34,35,36,37]. The flow diagram is depicted in Figure 1.

### 3.2. QUADAS-2 Risk of Bias Assessment

**Figure 2:**
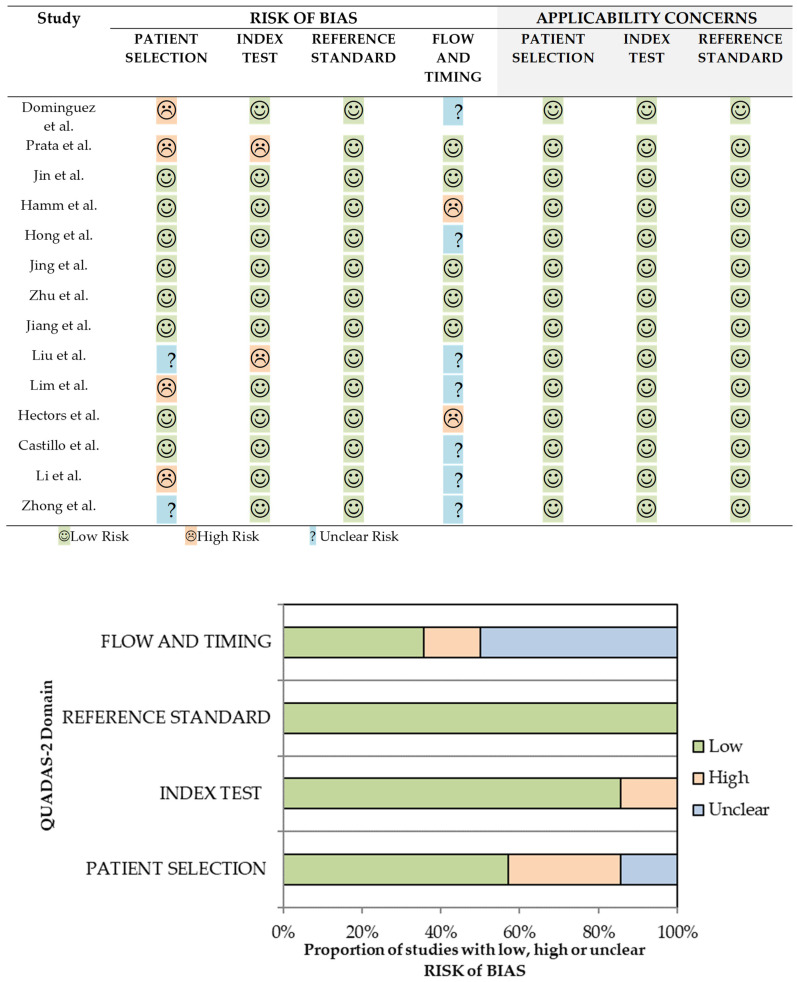
QUADAS-2 risk of bias and applicability concerns of the selected studies (**top**, [24,25,26,27,28,29,30,31,32,33,34,35,36,37]) and its corresponding graphical representation (**bottom**).

The results of the QUADAS-2 [22] assessment for each paper included are presented in Figure 2. A total of 4 out of 14 (29%) studies [26,29,30,31] had low risk of bias, while 7 out of 14 (50%) [24,25,27,32,33,34,36] had high risk of bias. The remaining three studies (21%) [28,35,37] had an unclear risk of bias. All papers had low applicability concerns.

Among the seven studies with high risk of bias, four of them had inadequate patient selection [24,25,33,36] because of inappropriate exclusion criteria (exclusion of lesions < 5 mm or advanced stages) [24,25,36] or case–control design [33], which might overestimate the results and conclusions. Moreover, there was also a high risk of bias in the index test in two studies [25,32] because the threshold used was not clearly specified to the best of our knowledge. There was also a high risk of bias in flow and timing in another two studies [27,34] because the period between the MRI and the prostate biopsy or radical prostatectomy exceeded three months in some cases. This might underestimate the risk of csPCa based on MRI interpretation because of a potential tumor progression during the waiting time. Finally, two studies (21%) had an unclear risk of bias in patient selection because the enrollment of the patients and/or exclusion criteria were not clear/reported [32,37].

### 3.3. Quality Assessment Based on RQS and CLAIM

The results of AI-specific quality assessment are first presented for the studies based on handcrafted radiomics using RQS [15], detailing the overall score and the specific results for each item in the checklist. Afterwards, the results of the studies based on deep radiomics are presented using CLAIM [23], highlighting the most important or controversial items of the checklist.

Eight studies (57%) used handcrafted radiomics for extracting image features [24,25,26,29,33,34,35]. The overall quality based on RQS [15] was low, with a median of 10.5 and interquartile range (IQR) of 2.5, out of a maximum of 36 points. All the studies reported the image quality protocol (item 1) and feature reduction or adjustment for multiple testing (item 4). However, none of them performed phantom studies (item 3), imaging at multiple timepoints (item 4), biological correlation (item 7), prospective studies (item 11), potential clinical applications (item 14), or cost-effectiveness analysis (item 15). Three studies (38%) performed multiple segmentations and/or feature robustness assessments (item 2) [26,29,36]. Multivariable analysis was performed in five studies [24,25,29,34,36], while the rest opted for an exclusive radiomic model [26,33,35]. Interestingly, four studies also made a comparison of the radiomic model or multivariable model with the PI-RADS classification (item 6) [24,25,29,35], albeit in only two was a statistical comparison between both given [24,29]. Moreover, only three studies conducted external validation (item 12) [26,29,35]. Discrimination statistics (item 9) were given in all the studies, although the confidence intervals were not reported in a single study [25]. Cut-off analysis (item 8) and calibration statistics (item 9) were reported in three [24,34,36] and two [26,29] studies, respectively. Lastly, a single study published the code used for creating the model [33].

Six studies (43%) used deep radiomics for extracting image features [27,28,30,31,32,37], and were assessed with CLAIM [23]. Among the different items in the methods section, none of the studies reported deidentification methods (item 12) nor how missing data were handled (item 13), although no study reported missing data, per se. The intended sample size and how it was determined (item 19) was also not specified in any of the studies, nor was robustness analysis (item 30). Although annotations were generally well explained, measurement of inter- and intrareader variability was not well reported. A detailed description of the model and its training (items 22 to 25) was generally well reported, although the initialization of model parameters (item 24) was only reported in one study [37], which used transfer learning. Metrics of model performance (item 28) were reported in all the studies, with the corresponding statistical measures of significance (item 29) except in a single study [28]. External validation (item 32) was carried out in half of the studies [27,28,31]. Importantly, a single study used explainability or interpretability methods (item 31) [27]. In the results section, two studies did not present the flow of participants in a diagram (item 33) [32,37]. The demographic and clinical characteristics in each partition (item 34) was partially, or not, performed in two studies [30,37]. Failure analysis of incorrectly classified cases was only properly conducted in a single study [31]. Finally, in the discussion and other information section, it is important to note that two studies used open code [27,31].

### 3.4. Study Characteristics

The characteristics of the selected studies are represented in five consecutive tables (Table 1, Table 2, Table 3, Table 4 and Table 5) following a continuous flow from the main clinical and demographic characteristics to specific details of the radiomic pipeline and, lastly, the metrics of the radiomic, clinical, or combined models developed in each paper.

Table 1 presents the main clinical, demographic, and radiological characteristics of the different cohorts included in the 14 selected studies [24,25,26,27,28,29,30,31,32,33,34,35,36,37]. The number of participants, origin (i.e., United States of America), and whether it was a unicentric or multicentric study is depicted, as well as the years in which the dataset was obtained. The amount of csPCa included in each paper and the number of lesions in the peripheral zone is also summarized. The MRI manufacturer and the magnetic field strength used for each cohort are represented, as well as the PI-RADS score of the lesions reported. Finally, the reference standard (prostate biopsy or radical prostatectomy) as well as the biopsy technique and the time between the MRI and the procedure are specified.

As seen in this table, most of the selected papers (10 of 14, 71%) used data from single institutions [24,25,27,28,30,31,32,34,36,37]. The number of men included ranged from 86 to 1230 patients, with a median of 294 and an IQR of 262 [24,25,26,27,28,29,30,31,32,33,34,35,36,37]. Two of the studies had more than 1000 patients [27,31]. All the studies were retrospective, in which eight (57%) had data collected for ≥4 years [24,27,28,29,31,32,34,37]. There was a general disbalance in the number of csPCa cases of the selected studies. Four studies had between 40 and 60% of csPCa cases [25,27,35,37], while the rest were more disbalanced, with 12% of csPCa cases as the lowest percentage [34] and 77% as the highest [24]. Almost half or more of the lesions were in the peripheral zone [24,26,27,28,29,30,31,32,33,35], with a median of 61% and IQR of 19%. The second most common location was the transitional zone, with a median of 33% and IQR of 7%. Five studies included a few lesions in other locations such as anterior fibromuscular stroma, central zone, or diffuse [27,28,29,32,35]. Four studies did not report the specific prostate location of the lesions [25,34,36,37].

Half of the selected studies included images from more than one MRI vendor (7 of 13, 54%) [26,28,29,31,33,34,35]. One study did not report the specific vendor [32] and another reported multiple vendors, but the brand was not specified [28]. Siemens and Philips were the most frequent vendors, present in 10 of the 12 studies (83%) [24,25,26,27,31,33,34,35,36,37]. A total of 12 of the 14 studies (86%) used a 3 Tesla as the magnetic field [24,26,27,28,29,30,31,32,33,34,36,37], and one used a 1.5 Tesla machine [25]. The remaining study used MRIs with both magnitudes [35]. Only two studies included some patients in which an endorectal coil was used [35,37]. A total of 10 of 14 studies specified the PI-RADS of the lesions included [24,25,26,27,28,29,31,32,33,34,35]. Seven (70%) included PI-RADS 3 and/or higher lesions [24,25,26,28,32,33,34], of which three included only PI-RADS 3 lesions [26,34,36].

Most of the studies (9 of 14, 64%) were based on prostate biopsy as the reference standard [24,25,26,27,30,31,33,34], and the remaining five (36%) were based on radical prostatectomy [28,29,32,35,37]. All of them had the procedure performed after the MRI, but half of the studies did not report the period between the MRI and the procedure. It ranged from four weeks to 12 months in the studies in which it was reported [25,26,27,29,30,31,33]. Transrectal ultrasound (US) was the preferred approach for performing the prostate biopsy in all the studies except in one case, which was not reported [27]. Five studies specified MRI/US fusion technique as the preferred choice [24,25,26,31,34] while only two preferred cognitive targeting [30,33]. The remaining two studies did not specify [27,36].

Table 2 describes the basic characteristics of the radiomic pipeline. As such, the technique used for extracting the features, either handcrafted radiomics or deep radiomics, is specified. The MRI sequences in which the radiomic features were obtained are given, as well as the origin (i.e., lesion segmentation or other parts of the prostate). Furthermore, several steps in a machine learning process such as image preprocessing, data imbalance or augmentation techniques, feature selection, and train/test split ratio are detailed. Finally, the algorithm used for constructing the model is also depicted.

Eight studies (57%) used handcrafted radiomics for extracting image features [24,25,26,29,33,34,35,36], while the remaining six (43%) relied on deep radiomics [27,28,30,31,32,37]. All the selected studies extracted the features from MRI T2 and/or ADC sequences, and in five (36%) [26,27,29,31,35], they were also extracted from high b-value DWI sequences. None of the studies extracted features from dynamic contrast-enhanced (DCE) sequences. Imaging features were extracted in all the selected studies from the lesion segmentations. Additional prostate segmentations were performed in four studies (29%) [27,28,29,31], although in only one study were they used for extracting image biomarkers [29]. The peripheral and transitional zones were also additionally segmented in one study [27]. All the segmentations were manually carried out except in one study, in which a predefined bounding box was created around the annotated lesions, and the prostate and prostate zones were automatically segmented with nn-Unet [27]. OsiriX was the most used software for performing the manual segmentations, which was used in three studies (21%) [25,32,37]. Slicer [24,33] and ITK-SNAP [26,30] were the second most used software tools. One study did not report the tool [34]. The radiologist experience was specified in all but one study [37], ranging from 3 to more than 10 years of experience. In three studies [25,33,34], the segmentations and/or relabeling of the lesions were performed by a single radiologist.

Image preprocessing was reported and performed in 11 studies (79%) [26,27,28,29,30,31,32,34,35,36,37]. One study reported that image preprocessing was not needed since all the images were acquired with the same protocol and resolution [24]. Intensity normalization and resampling were the most frequently performed. Image registration was reported in four studies (29%) [26,30,31,32], in which the images were spatially matched using Elastix software in two of them [26,31]. In contrast, data imbalance techniques and data augmentation were barely reported. The former was reported in five studies (36%) [26,28,34,35,37]. The most common method was synthetic minority oversampling technique (SMOTE) [26,34]. In one study it was regarded as not necessary [37]. Data augmentation was performed in five studies [27,29,31,35,37].

Among the eight studies that used handcrafted radiomics [24,25,26,29,33,34,35,36], PyRadiomics was the most used library for extracting the imaging features [24,29,33,34]. Feature robustness was assessed in three of them (38%) [26,29,36]. Feature normalization was also reported in three studies (38%) [24,26,35], with z-Score as the method used. Feature harmonization was reported in one study [35], which used ComBat. Finally, feature selection was performed in the eight studies [24,25,26,29,33,34,35,36], with different algorithms specified in Table 2. Train–test split was the preferred method for training the model in 12 of the selected studies [24,26,27,28,29,30,31,32,34,35,36,37], with 80/20% being the most common partition. The remaining two studies performed a cross-validation [25,33]. The algorithm used for creating the model varied between studies, but classic machine learning algorithms were used for handcrafted radiomics studies, and deep neural networks were used for deep radiomic studies.

Table 3 and Table 4 depict the overall results of the radiomic models, divided into studies that use handcrafted radiomics (in Table 3) or deep radiomics (in Table 4). In both tables, the validation strategy (i.e., internal or external validation) and the specific analysis (i.e., per index lesion) are detailed. The AUC, sensitivity, and specificity of the best radiomic model for csPCa prediction are also given, alongside the MRI sequences in which the image features were extracted that proved to be the most relevant for the prediction. For comparison, the metrics of the PI-RADS evaluation are also depicted if it was assessed, with the threshold considered as csPCa in such cases (i.e., csPCa is considered if PI-RADS ≥ 4).

In the studies based on handcrafted radiomics [24,25,26,29,33,34,35,36], index lesion was the preferred analysis except in one case in which the analysis was based on all the lesions [33]. Three of the eight studies (38%) performed an external validation [26,29,35]. The AUC was reported in all the studies and ranged from 0.72 to 0.98 for index lesions in the internal validation. The results for the external validation sets were similar to the ones obtained in the internal validation, being 0.75 and 0.95. Sensitivity and specificity were reported in five of the eight handcrafted radiomics studies [26,29,35,36]. In the studies based on deep radiomics [27,28,30,31,32,37], the preferred analysis was more diverse since it included the index lesion and all the lesions, as well as a sextant-level analysis in one study [30]. Three of the six studies (50%) conducted an external validation [27,28,31], albeit two [27,31] were based on the PROSTATEx public dataset [38]. The AUC was reported in all but one study [30], which ranged from 0.73 to 0.85 for index lesions and 0.73 to 0.89 for all lesions in internal validation. The values were 0.63 for index lesions and 0.86 and 0.87 for all lesions in the external validation.

The PI-RADS performance was evaluated in half of the studies [24,25,29,30,31,32,35,37], in which five reported the AUC, sensitivity, and specificity [29,31,32,35,37]. The statistical comparison between the radiomic model and PI-RADS was assessed in four studies [30,31,32,37]. Zhu et al. [30] reported no significant differences in sensitivity between both models (considering PI-RADS ≥ 3 as csPCa) at index lesion, sextant-level, and all-lesions-level analysis. Liu et al. [32] reported a similar performance between both models (considering PI-RADS ≥ 4 as csPCa) at index lesion based on AUC, but the radiomic model performed significantly better than PI-RADS in all lesion-level analysis. Zhong et al. [37] reported no significant differences between both models (considering PI-RADS ≥ 4 as csPCa) based on AUC at all lesion levels. In contrast, Jiang et al. [31] reported a significantly better performance of the PI-RADS model (considering PI-RADS ≥ 3 as csPCa) in the internal validation and similar in external validation, based on AUC.

Table 5 assesses other tested models such as clinical models and/or combined models (clinical variables with radiomic features), and it is displayed in a similar way to Table 3 and Table 4 with the validation strategy, specific analysis, and metrics detailed.

In six studies [24,25,26,29,34,36], PSA density (PSA-D), clinical models, and combined models were also assessed. Dominguez et al. [24] reported a significantly better performance of the combined model in comparison to PI-RADS (cut-off not reported) and PSA-D in the cross-validation. Jing et al. [29] also reported a significantly better performance of the combined model in comparison to PI-RADS (cut-off not reported) in internal and external validation. Li et al. [36] showed no significant differences between the radiomic model and combined models, but both were better than the clinical model.

## 4. Discussion

This systematic review evaluated the current evidence of deep and handcrafted radiomics models in distinguishing csPCa from iPCa and benign lesions in prostate MRIs assessed with PI-RADS v2 and/or v2.1. The selected studies demonstrated good performance for index lesion classification, with handcrafted radiomics models achieving AUCs ranging from 0.72 to 0.98, and deep radiomics models achieving AUCs from 0.73 to 0.85. A meta-analysis was not conducted due to the significant heterogeneity in the datasets, methodologies, model development, and validation of the selected studies, preventing definitive conclusions. Nevertheless, there is no clear difference between the performance of both approaches, nor between internal and external validations, consistent with other reviews [39]. A meta-analysis published in 2019 favored handcrafted over deep radiomics models [40], although the authors noted that the low number of participants in the selected studies might have favored handcrafted methods. Developing deep learning models to achieve expert performance requires large amounts of data [41], so we believe that deep radiomic models will surpass handcrafted ones in the future as recent studies incorporate progressively more data. A recent review published in 2022 slightly favored deep radiomic methods over traditional ones, despite not being a meta-analysis [42].

The substantial heterogeneity of the included studies is also observed in other similar reviews [39,40,42,43,44]. Specific eligibility criteria were designed to mitigate this limitation. First, studies with preprocedure MRI were included to avoid misinterpretation due to hemorrhage, which can affect radiologist judgment and induce bias [45]. Second, only studies using PI-RADS v2 and/or v2.1 for lesion assignment were included, as these provide better interpretability than PI-RADS v1 or Likert score [46,47]. Third, targeted biopsies (combined or not with systematic biopsies) or radical prostatectomies were the chosen reference standards. Exclusive systematic biopsies were excluded due to their inferior performance compared to targeted biopsies [48], which has been a source of heterogeneity in past reviews [40,42]. Moreover, mixing targeted biopsies and radical prostatectomies was avoided to homogenize the data, despite no clear pathological upgrading of radical prostatectomy compared to targeted prostate biopsy [49]. A recent study showed no differences in model performance based on reference standard [50], but further assessment is needed. Studies involving men in active surveillance or with prior prostate cancer treatment were excluded to prevent bias towards higher-risk patients. Finally, studies based on public repository datasets were excluded to ensure multicentric and larger studies, addressing issues highlighted in past reviews [40,42]. However, public repositories will be crucial in the future due to the current lack of sufficient multicentric data. Significant efforts are being made in this area [51], which are beyond the scope of this review. Despite these efforts, significant heterogeneity and restrictions were found in the data extracted and the quality analysis using QUADAS-2 [22], RQS [15], and CLAIM [23] tools, which will be discussed in the following paragraphs, along with recommendations for future studies.

First, there were several methodological constraints that might introduce bias into radiomics models, starting with data issues. Most of the studies were based on single-center datasets and exhibited data imbalance, with an overrepresentation of csPCa cases and a predominance of peripheral zone lesions. Data imbalance and lack of multicentric datasets are common problems in AI in medical imaging, which can introduce bias [52,53]. Although this is intrinsic to the collected data and difficult to overcome in healthcare due to data scarcity, few of the selected studies applied techniques to address data imbalance [26,28,34,35,37] or used data augmentation techniques [27,30,31,35,37]. Moreover, some studies excluded lesions smaller than 5 mm or advanced stages, introducing a bias by reducing false positives and excluding high-risk patients [24,25,36]. This reduces data representativity and may lead to bias, contributing to high-risk assessments in the QUADAS-2 evaluation [22]. Similarly, most studies used images from only one or two different MRI vendors and a magnetic field strength of 3T, which also reduces data representativity. Some nonselected studies reported no significant differences in performance based on magnetic field strength or MRI vendor [50,54,55], but further assessment is needed. Additionally, despite efforts to mitigate bias due to the chosen reference standard, few studies reported the time between the MRI and the procedure, or exceeded three months, contributing to unclear and high-risk bias, respectively [24,27,28,32,33,34,35,36,37]. It is also important to emphasize the interobserver variability between pathologists when assessing the Gleason Score, so the pathologist’s experience should be reported [56].

Secondly, the review highlighted sources of bias in the radiomic pipeline. One of the most notable was the limited data on interobserver/inter-reader agreement when segmenting lesions, as noted in the RQS [15] and CLAIM [23] evaluations. Manual segmentations performed by multiple radiologists introduce heterogeneity and influence model performance. Although radiologist experience was specified in all but one paper [37], there was limited evaluation of interobserver/inter-reader variability in most cases. Similarly, in studies based on handcrafted radiomics, feature robustness was rarely assessed. This is important because radiomic features have low reproducibility and repeatability [57,58], introducing clear bias. In contrast, feature selection was performed in all the handcrafted radiomic studies, and the top selected features were reported except in two studies [33,35]. Image preprocessing was also well defined in most of the included studies, allowing reproducibility. None of the studies extracted features from dynamic contrast-enhanced (DCE) sequences. There has been a progressive decline in the number of studies that extract features from DCE in favor of T2 and/or ADC, as noted in similar reviews [39,42]. There is no clear added value in comparison to T2 and ADC [39]. All the studies extracted features from T2 and/or ADC sequences, and four of them from high-b value DWI [26,27,31,35]. While high-b values are better than low-b values for detecting PCa [59], there is controversy about the added value of DWI if features are already extracted from ADC, leading to potential bias [60]. In the studies that included both sequences, there was no clear drop in performance, but further assessment is needed [26,31,35].

Thirdly, there were important limitations in the training/validation of the models. The most significant one is the lack of external validation cohorts. Past similar reviews also highlighted this problem [39,40,42,43,44], which limits model applicability and robustness [61]. Six studies used external validation sets [26,27,28,29,31,35], but two of them were from public repositories [25,29]. Calibration studies should also be performed in external cohorts, but only two studies reported them [26,29]. There were also other constraints regarding the training/validation of the models, such as no mention of the minimum sample size needed to detect a clinically significant effect size and make comparisons [62], as well as poor reporting of how thresholds were chosen or reported. Moreover, all the studies were retrospective, which inherently induces bias due to the design and limited data. Prospective studies are needed to better assess the potential of AI models in clinical practice. Efforts are being made in this regard, and some prospective studies are being published with encouraging results [63]. Additionally, open-source code should be used to favor transparency and reproducibility, as specified in the RQS [15] and CLAIM [23] tools. Only three studies used open-source code [27,31,33]. Potential clinical applications should also be discussed, such as using the models as a second reader [64]. Explainability methods are also required to facilitate clinical implementation. In this review, only one study used interpretability methods [27].

Lastly, other objectives of this review were to compare radiomic models, radiologists, and multivariable models. This issue has been noted in past reviews [39,40] since there is a lack of comparisons between AI-based models and current clinical practice [65]. In fact, only four studies conducted a statistical comparison between the radiomic model and the PI-RADS classification [30,31,32,37], using PI-RADS ≥ 3 or ≥4 as the thresholds for detecting csPCa. Overall, there was no clear difference between the performance of PI-RADS and the models. Liu et al. [32] reported significantly better performance of the radiomic model at all lesion levels but not at the index lesion level. Jiang et al. [31] reported a significantly better performance of PI-RADS in the internal validation set but found no differences at external validation. Future studies should assess this issue to favor clinical implementation, as well as comparing the performance based on radiologist expertise. Hamm et al. [27] reported better performance of nonexpert readers when using the AI assistance, especially in PI-RADS 3 lesions, which represents a challenge due to the overdiagnosis of iPCa [8]. It is important to consider that there is also inherent inter-reader variability in MRI interpretation with PI-RADS system among radiologists [12,13], as well as limitations of the PI-RADS v2.1 [11], but these limitations are beyond the scope of this review. Four studies created multivariable models that incorporated clinical variables (including PI-RADS in some cases) [24,25,29,36]. Dominguez et al. [24] and Jing et al. [29] reported significantly better performance of the combined model than the PI-RADS. Future studies are needed to better assess the role of radiomics in combined models to improve the current standard based on PI-RADS.

In the light of the above, we offer the following recommendations for future studies to assess the constraints and heterogeneity and encourage clinical applicability: (i) large and multicentric datasets with representative and balanced data for the clinical aim of the model should be used; (ii) clear inclusion and exclusion criteria should be well specified, avoiding criteria that make nonrepresentative or biased data such as exclusion of advanced stages; (iii) detailed methodology, preferably following published AI guidelines for medical imaging (such as CLAIM [23]); (iv) robust reference standard, such as targeted biopsy or radical prostatectomy; (v) prospective design is desired; (vi) assessment of interobserver/inter-reader variability in manual segmentations, as well as feature robustness; (vii) detailed statistical methods, including sample size calculation and appropriate discrimination metrics with statistical significance and information about selected thresholds; (viii) validation on external datasets; (ix) open source and explainability methods are encouraged; (x) comparison of the model with current PI-RADS version, as well as development of combined models with clinical variables (such as PSA-D, DRE or others).

This review had some limitations. First, the publication bias favors studies with good performance that might overestimate the results. Second, relevant studies published after the deadline of the review might have been missed. Third, the specific eligibility criteria might have discharged relevant studies in which the methodology was not properly defined. Lastly, no direct comparisons and analysis were possible due to the heterogeneity of the data.

## 5. Conclusions

This systematic review denotes promising results of radiomic models in the prediction of csPCa in the included studies. However, the quality evaluation highlights significant heterogeneity and constraints that limit the clinical application of these models. This includes limited data representativity and methodological errors in the radiomic pipeline such as proper evaluation of interobserver/inter-reader variability or feature robustness, as well as a lack of prospective studies and external validation to evaluate the real performance outside the internal dataset. Furthermore, more efforts are needed to compare these models with radiologists and the integration of radiomics in combined models with other clinical variables. Future studies should tackle these problems to better understand the potential of radiomics in this field and ensure proper implementation in routine clinical practice.

## Figures and Tables

**Table 1:** Basic demographics, MRI/PI-RADS characteristics, and reference standard details of the selected studies.

Reference, Year	Data Source, n, Country	Dataset Year/s	csPCa, n (%)	PZ, n (%)	MRI Vendor, Tesla	PI-RADS lesions	Reference Standard, MRI to Procedure	Biopsy Technique
Dominguez et al. 2023 [24]	Single-center, 86, Chile	2017–2021	66 (0.77)	81 (0.94)	Philips, 3	≥3	PB, NR	MRI/US fusion, TRUS
Prata et al. 2023 [25]	Single-center, 91, Italy	2019–2020	39 (0.43)	NR	Siemens, 1.5	≥3	PB, within 4 weeks	MRI/US fusion, TRUS
Jin et al. 2023 [26]	Multicenter, 463, China	2018–2019	100 (0.22)	216 (0.47)	Siemens/Philips, 3	3	PB, within 4 weeks	MRI/US fusion, TRUS
Hamm et al. 2023 ^a^ [27]	Single-center, 1224, GER	2012–2017	595 (0.49)	1935 (0.59) ^b^	Siemens, 3	≥1	PB, within 6 months	NR
Hong et al. 2023 ^a^ [28]	Single-center, 171, Korea	2018–2022	40 (0.37)	81 (0.47)	Multivendor, 3	≥3	RP, NR	NA
Jing et al. 2022 [29]	Multicenter, 389, China	2016–2021	270 (0.69)	190 (0.49)	GE/UI, 3	≥2	RP, within 12 weeks	NA
Zhu et al. 2022 [30]	Single-center, 347, China	2017–2020	235 (0.68)	212 (0.68) ^c^	GE, 3	NR	PB, within 12 weeks	CT, TRUS
Jiang et al. 2022 ^a^ [31]	Single-center, 1230, China	2012–2019	856 (0.63) ^b^	853 (0.63) ^b^	Siemens/UI, 3	≥1	PB, within 4 weeks	MRI/US fusion, TRUS
Liu et al. 2021 [32]	Single-center, 402, USA	2010–2018	303 (0.75) ^b^	364 (0.78) ^b^	NR, 3	≥3	RP, NR	NA
Lim et al. 2021 [33]	Multicenter, 158, Canada	2015–2018	29 (0.18) ^b^	79 (0.49) ^b^	Siemens/GE, 3	3	PB, NR	CT, TRUS
Hectors et al. 2021 [34]	Single-center, 240, USA	2015–2020	28 (0.12)	NR	Siemens/GE, 3	3	PB, within 12 months	MRI/US fusion, TRUS
Castillo et al. 2021 [35]	Multicenter, 107, NL	2011–2014	112 (0.55) ^b^	137 (0.67) ^b^	GE/Siemens/Philips, 3/1.5	≥1	RP, NR	NA
Li et al. 2020 [36]	Single-center, 381, China	2014–2017	142 (0.37)	NR	Philips, 3	NR	PB, NR	TRUS
Zhong et al. 2019 [37]	Single-center, 140, USA	2010–2016	105 (0.49) ^b^	NR	Siemens, 3	NR	RP, NR	NA

csPCa = clinically significant prostate cancer, CT = cognitive targeting, GE = General Electrics, GER = Germany, MRI = magnetic resonance imaging, NA = not applicable, NL = Netherlands, NR = not reported, PB = prostate biopsy, PZ = peripheral zone, PI-RADS = Prostate Imaging Reporting and Data System, RP = radical prostatectomy, TRUS = trans-rectal ultrasound, UI = United Imaging, US = ultrasound, USA = United States of America. ^a^ Data from external validation sets are not included in the description (see reference for further details); ^b^ Data referred to as annotated lesions; ^c^ Data are for csPCa lesions.

**Table 2:** Main characteristics of the machine learning process of the selected studies.

Reference, Year	Sequences	Segmentation	Feature Extraction	Image Preprocessing	Data Imbalance techniques, Data Augmentation	Feature Selection	Train/Test (%) ^b^	Algorithm
Dominguez et al. 2023 [24]	T2, ADC	Lesion	Shape, FO, HTF	Not performed	NR	RFE	80 (CV)/20	LR
Prata et al. 2023 [25]	T2, ADC	Lesion	FO, HTF, BLP	NR	NR	Wrapper (RF)	CV	RF
Jin et al. 2023 [26]	T2, ADC, DWI (b2000)	Lesion	FO, HTF, wavelet features	IN, grey-level quantization, resampling, IR	SMOTE, NR	ANOVA	70/30	SVM
Hamm et al. 2023 [27]	T2, ADC, DWI (high-b value)	Lesion, prostate, PZ, TZ	Deep radiomics	IN, resampling, lesion cropping	NR, Yes	NA	80 (CV)/20	Visual Geometry Group Net-based CNN
Hong et al. 2023 [28]	ADC	Lesion, prostate	Deep radiomics	IN, resizing, prostate cropping, cut-off filtering	Image allocation, NR	NA	80/20	DenseNet 201
Jing et al. 2022 [29]	T2, DWI (b1500)	Lesion, prostate	Shape, FO, HTF, higher-order features	IN, Resampling	NR	Variance threshold algorithm, Select K-best, LASSO	70/30	LR
Zhu et al. 2022 [30]	T2, ADC	Lesion	Deep radiomics	IN, resampling, prostate cropping, IR	NR, Yes	NA	60/40	Res-UNet
Jiang et al. 2022 [31]	T2, DWI (b1500), ADC	Lesion, prostate	Deep radiomics	IN, resampling, prostate cropping, IR	NR, Yes	NA	66.6/33.3	Attention-Gated TrumpetNet
Liu et al. 2021 [32]	T2, ADC	Lesion	Deep radiomics	IN, lesion cropping, IR	NR	NA	70/30	3D GLCM extractor + CNN
Lim et al. 2021 [33]	T2, ADC	Lesion	Shape, FO, HTF	NR	NR	Mann–Whitney U-test	CV	XGBoost
Hectors et al. 2021 [34]	T2	Lesion	Shape, FO, HTF	IN, grey-level quantization, resampling	SMOTE, NR	RF	80 (CV)/20	RF, LR
Castillo et al. 2021 [35]	T2, DWI (highest-b value), ADC	Lesion	Shape, FO, HTF, higher-order features	Resampling	WORC Workflow ^a^	WORC Workflow ^a^	80 (CV)/20	WORC Workflow ^a^
Li et al. 2020 [36]	T2, ADC	Lesion	FO, HTF	IN, grey-level quantization, resampling	NR	mRMR, LASSO	60/40	LR
Zhong et al. 2019 [37]	T2, ADC	Lesion	Deep radiomics	IN, resizing, lesion cropping	Not necessary, Yes	NA	80/20	ResNet with TL

BLP = binary local pattern, CNN = convolutional neural network, CV = cross-validation, FO = first order, GLCM = gray-level co-occurrence matrix, HTF = handcrafted texture features, IN = image normalization, IR = image registration, LASSO = least absolute shrinkage and selection operator, LR = logistic regression, mRMR = minimum redundancy maximum relevance, NR = not reported, NA = not applicable, PZ = peripheral zone, RFE = recursive feature elimination, RF = random forest, SMOTE = synthetic minority oversampling technique, SVM = support vector machine, TZ = transitional zone. ^a^ Uses a radiomics workflow called Workflow for Optimal Radiomics Classification (WORC), which includes different workflow processes (see reference for further details). ^b^ Presented as % of the data selected for the training and test partitions. CV stands for cross-validation performed in the training set.

**Table 3:** Analysis, validation, and results for csPCa prediction in the selected studies based on handcrafted radiomics as the feature extraction method.

Reference, Year	Analysis	Validation	Sequence for the Best Model	Best Radiomic Model [CI, 95%] ^a^			PI-RADS Cut-Off	PI-RADS Model [CI, 95%] ^a^		
				AUC	Sensitivity	Specificity		AUC	Sensitivity	Specificity
Dominguez et al. 2023 [24]	Index	CV//Hold-out set	ADC	0.81 [0.56–0.94]//0.71	NR	NR	NR	0.66 [0.57–0.74]//NR	NR	NR
Prata et al. 2023 [25]	Index	CV	ADC	0.77	NR	NR	NR	0.68	NR	NR
Jin et al. 2023 [26]	Index	Hold-out set//External (1 set)	T2 + ADC + DWI (b2000)	0.80//0.80	0.80//0.73	0.65//0.92	NA	NA	NA	NA
Jing et al. 2022 [29]	Index	Hold-out set//External (2 sets)	T2 (prostate) + DWI b1500 (lesion)	0.96 [0.90, 1.00]//0.95 [0.87, 1.00]//0.94 [0.90, 0.99] ^b^	0.95//0.98//0.86 ^b^	0.94//0.86//0.91 ^b^	NR	0.84 [0.74, 0.95]//0.82 [0.72, 0.93]//0.80 [0.71, 0.88]	0.98//0.98//0.50	0.56//0.52//0.94
Lim et al. 2021 [33]	All	CV	ADC	0.68 [0.65–0.72]	NR	NR	NA	NA	NR	NR
Hectors et al. 2021 [34]	Index	Hold-out set	T2	0.76 [0.60–0.92]	0.75	0.8	NA	NA	NA	NA
Castillo et al. 2021 [35]	Index	CV//External	T2 + ADC + DWI (highest-b value)	0.72 [0.64, 0.79]//0.75	0.76 [0.66, 0.89]//0.88	0.55 [0.44, 0.66]//0.63	≥3	0.50//0.44 (2 radiologists, External Validation)	0.76//0.88	0.25//0
Li et al. 2020 [36]	Index	Hold-out set	T2 + ADC	0.98 [0.97–1.00]	0.95	0.87	NA	NA	NA	NA

All = all lesions, AUC = area under the curve, CI = confidence interval, csPCa = clinically significant prostate cancer, CV = cross-validation, Index = index lesion, NA = not applicable, NR = not reported, PI-RADS = Prostate Imaging Reporting and Data System. ^a^ Data are expressed in the corresponding metric and the CI, 95% for each validation method separated by//. If the CI is not included, it means that it was not reported in the study. ^b^ The combined model (radiomic model + PI-RADS) is included since there are no data for the radiomic model.

**Table 4:** Analysis, validation, and results for csPCa prediction in the selected studies based on deep radiomics as the feature extraction method.

Reference, Year	Analysis	Validation	Sequence for the Best Model	Best Radiomic Model [CI, 95%] ^a^			PI-RADS Cut-Off	PI-RADS Model [CI, 95%] ^a^		
				AUC	Sensitivity	Specificity		AUC	Sensitivity	Specificity
Hamm et al. 2023 [27]	All	Hold-out set//External (PROSTATEx)	T2 + ADC + DWI (high-b value)	0.89 [0.85, 0.93]//0.87 [0.81, 0.93]	0.77 [0.69, 0.85]//0.90 [0.83, 0.97]	0.89 [0.84, 0.95]//0.85 [0.80, 0.90]	NA	NA	NA	NA
	Index			0.78//NR	0.98 [0.95, 1.00] ^b^//NR	NR				
Hong et al. 2023 [28]	Index	Hold-out set//External (1 set)	ADC	NR//0.63	0.72//0.84	0.74//0.48	NA	NA	NA	NA
Zhu et al. 2022 [30]	All	Hold-out set	T2 + ADC	NR	0.96 [0.89, 0.99]	NR	≥3	NR	0.94 [0.87, 0.98]	NR
	Sextant			NR	0.96 [0.90, 0.99]	0.92 [0.89, 0.93]		NR	0.93 [0.87, 0.97]	0.92 [0.90, 0.94]
	Index			NR	0.99 [0.92, 0.99]	0.65 [0.53, 0.76]		NR	0.99 [0.92, 0.99]	0.66 [0.54, 0.77]
Jiang et al. 2022 [31]	All	Hold-out set//External (PROSTATEx)	T2 + ADC + DWI (b1500)	0.85 [0.81, 0.88]//0.86 [0.81, 0.91]	0.93//0.87	0.5//0.66	≥3	0.92 [0.89, 0.95]//0.86 [0.80, 0.90]	0.94//0.77	0.79//0.87
Liu et al. 2021 [32]	All	Hold-out set	T2 + ADC	0.85 [0.79, 0.91]	0.90 [0.83, 0.96]	0.70 [0.59, 0.82]	≥4	0.73 [0.65, 0.80]	0.83 [0.75, 0.92]	0.47 [0.35, 0.59]
	Index			0.73 [0.59, 0.88]	0.90 [0.83, 0.96]	0.47 [0.21, 0.72]		0.65 [0.52, 0.78]	0.83 [0.75, 0.91]	0.27 [0.04, 0.72]
Zhong et al. 2019 [37]	All	Hold-out set	T2 + ADC	0.73 [0.58, 0.88]	0.64	0.8	≥4	0.71 [0.58, 0.87]	0.86	0.48

All = all lesions, AUC = area under the curve, CI = confidence interval, csPCa = clinically significant prostate cancer, CV = cross-validation, Index = index lesion, NA = not applicable, NR = not reported, PI-RADS = Prostate Imaging Reporting and Data System. ^a^ Data are expressed in the corresponding metric and the CI, 95% for each validation method separated by//. If the CI is not included it means that it was not reported in the study. ^b^ At 2 false-positive rate.

**Table 5:** Analysis, validation, and results for csPCa prediction in the selected studies based on clinical and combined models.

Reference, Year	Analysis	Validation	PSA-D [CI, 95%]^a^			Clinical Model [CI, 95%] ^a^			Combined Model [CI, 95%] ^a^		
			AUC	Sensitivity	Specificity	AUC	Sensitivity	Specificity	AUC	Sensitivity	Specificity
Dominguez et al. 2023 [24]	Index	CV//Hold-out set	0.77 [0.66–0.87]//NR	NR	NR	0.76 [0.62–0.87]//0.80 (PV-MR, PSA, PSA-D)	NR	NR	0.91 [0.76–0.99]//0.80 (Clinical Model and Radiomic Model)	NR	NR
Prata et al. 2023 [25]	Index	Hold-out set	NA	NA	NA	0.69 (DRE, PI-RADS)	NR	NR	0.80 (DRE, PI-RADS and Radiomic Model)	0.915	0.844
Jin et al. 2023 [26]	Index	Hold-out set//External (1 set)	0.71//0.69	0.84//0.77	0.60//0.62	NA	NA	NA	N/A	NA	NA
Jing et al. 2022 [29]	Index	Hold-out set//External (2 sets)	NA	NA	NA	NA	N/A	N/A	0.96 [0.90, 1.00]//0.95 [0.87, 1.00]//0.94 [0.90, 0.99] (Radiomic Model + PI-RADS)	0.952//0.978//0.861	0.944//0.857//0.907
Hectors et al. 2021 [34]	Index	Hold-out set	0.61 [0.41, 0.80]	0.72	0.52	NA	NA	NA	NA	NA	NA
Li et al. 2020 [36]	Index	Hold-out set	NA	NA	NA	0.79 [0.70–0.88] (Age, PSA, PSA-D)	0.76	0.74	0.98 [0.97–1.00] (Age, PSA, PSA-D and Radiomic Model)	0.82	0.97

AUC = area under the curve, CI = confidence intervals, CV = cross-validation, csPCa = clinically significant prostate cancer, DRE = digital rectal examination, Index = index lesion, NA = not applicable, NR = not reported, PI-RADS = Prostate Imaging Reporting and Data System, PSA = prostate-specific antigen, PSA-D = prostate-specific antigen density, PV-MR = prostate volume calculated with magnetic resonance. ^a^ Data are expressed in the corresponding metric and the CI, 95% for each validation method separated by//. If the CI is not included it means that it was not reported in the study.

## Supplementary Materials

The following supporting information can be downloaded at: https://www.mdpi.com/article/10.3390/cancers16172951/s1, Supplementary Table S1: Schematic representation of the eligible criteria extracted from the Population, Intervention, Comparator, Outcome (PICO) framework. Supplementary File S2: An explanation of the relevant data extracted from each selected study.
