# Supplementary material for: The Role of Radiomics in the Prediction of Clinically Significant Prostate Cancer in the PI-RADS v2 and v2.1 Era: A Systematic Review
Source: Cancers (Basel). 2024 Aug 24;16(17):2951. doi: 10.3390/cancers16172951 (PMC11393977; doi:10.3390/cancers16172951)
Supplement: Supplementary file 1 [file cancers-16-02951-s001.zip › cancers-3138007-supplementary.pdf]

Supplementary Information

S1. PICO framework and breakdown. PCa = Prostate Cancer; mpMRI = multiparametric MRI; bpMRI = biparametric MRI; csPCa = Clinically Significant Prostate Cancer; ISUP = International Society of Urogenital Pathology.

PICO	DEFINITION
**P** (Population)	Men with **suspected PCa**<br>+<br>**mpMRI/bpMRI with PI-RADS v2/2.1 score** of reported lesions<br>+<br>**Targeted +/- systematic biopsies or radical prostatectomy performed after the mpMRI or bpMRI**
**I** (Intervention)	**Handcrafted or Deep Radiomics analysis** of the lesions depicted in the MRI
**C** (Comparator)	**Histopathological confirmation of PCa** obtained from **radical prostatectomies or targeted +/- systematic biopsies**<br>+<br>csPCa defined as ISUP grade group > 1, and iPCa as grade group 1
**O** (Outcome)	**Uni- or multivariate risk model to predict csPCa from iPCa/benign lesions,** with a measurable metric

S2. Data extracted from the selected studies.

For each study the data source (single or multi-center), number of participants and the corresponding country was extracted. The period in which the magnetic resonance imaging (MRI) were done was also annotated, as well as the modality, multiparametric or biparametric MRI, Tesla magnitude and the coil used. The specific MRI manufacturer was also regarded as important. The PI-RADS v2/v2.1 score of the included lesions was also collected, as well as the number of clinically significant prostate cancer (csPCa), indolent prostate cancer (iPCa) and benign lesions (or non-csPCa if not specified). The specific location of the lesions in the prostatic gland was also extracted. The type of segmentation (manual, semi-automatic or automatic) and the preferred software was included, as well as the specific segmentations (lesion, prostate or prostate zones). The experience of the radiologist(s) who performed the segmentations or reassessment of PI-RADS lesions was also considered.

The ground-truth was collected (prostate biopsy or radical prostatectomy), as well as the biopsy technique and approach (transrectal ultrasound-guided, cognitive targeting...). The time between the MRI and the procedure was also annotated when available. The amount of biopsy cores, as well as urologist and/or pathologists experience was also collected when available.

For the studies based on handcrafted radiomics the category and number of extracted features (first order, high order...) were extracted, as well as other useful information (elimination of higher correlated features, feature harmonization, feature normalization, feature robustness, feature selection). Other information extracted (including studies based on deep radiomics) was: image preprocessing, image registration, data imbalance techniques and data augmentation. The algorithm and the library used for creating the model was also annotated. The MRI sequences used for creating the radiomic model were also collected. Non-radiomic models (i.e. PI-RADS) were also taken into account.

The train/test distribution, as well as the specific validation technique (including the use of cross-validation) and analysis performed was regarded as important. Finally, the metrics of the different models were collected, specifically the area under the curve, sensitivity and specificity. Comparisons between the models (i.e. radiomic model vs PI-RADS) were also considered.
